# Referrals for proliferative diabetic retinopathy from two UK diabetic retinopathy screening services: a 10-year analysis of visual outcomes, requirement for vitrectomy, and mortality

**DOI:** 10.1038/s41433-024-03078-1

**Published:** 2024-04-23

**Authors:** Salman Naveed Sadiq, Chan Ning Lee, Ben Charmer, Emily Jones, Maged S. Habib, Maria T. Sandinha, Ticiana Criddle, David H. W. Steel

**Affiliations:** 1 Sunderland Eye Infirmary, Queen Alexandra Road, Sunderland, SR2 9HP UK; 2 St. Paul’s Eye Unit, The Royal Liverpool University Hospital, Liverpool, UK; 3 Biosciences Institute, Newcastle University, Newcastle Upon Tyne, UK; 4 Department of Eye and Vision Science, Institute of Ageing and Chronic Disease, University of Liverpool, Liverpool, UK

**Keywords:** Outcomes research, Risk factors

## Abstract

**Background/objectives:**

To determine long-term outcomes of patients referred with proliferative diabetic retinopathy (PDR) from diabetic eye screening programmes (DESP) to tertiary care centres in the United Kingdom (UK).

**Methods:**

Retrospective multicentre study of patients referred from two DESPs in the UK over a 36-month period (2007–9) and followed-up for 10 years. Critical outcomes included severe vision loss (SVL) and the need for vitrectomy. Other outcomes assessed included moderate vision loss (MVL), and patient survival time. Univariate and multiple variable Cox proportional hazards regressions were used to analyse survival outcomes.

**Results:**

212 eyes of 150 patients were referred with a diagnosis of PDR. 109 eyes of 72 patients were confirmed to have active PDR and included in the study. 61% of patients had low-risk PDR, while 39% exhibited high-risk features in at least one eye. Eight (7.3%) eyes developed SVL and 16 (14.7%) MVL during follow up. Vitrectomy was required in 24% (95% CI: 15 to 31%) of all PDR eyes and was most commonly performed for vitreous haemorrhage (65%). The 10-year survival in all PDR patients was 76% (95% CI: 63 to 85%) with the mean time to death for all deceased patients being 5.4 ± 3.6 years.

On multivariable analysis, only age was found to have a significant association with the survival of patients with PDR.

**Conclusions:**

During the 10 year follow up SVL was uncommon, but MVL occurred in almost one-fifth of the eyes. Approximately 1 in 4 eyes required vitrectomy, highlighting its significance in patient management.

## Introduction

Proliferative diabetic retinopathy (PDR) is a leading cause of blindness in the working-age population [1, 2]. According to the International Diabetes Federation (IDF), the estimated number of people worldwide living with diabetes is around 463 million, and it is projected to increase by nearly 50.0% in the next 25 years reaching over 700 million (1 in 8 adults) by 2045 [3]. This will, in turn, lead to a substantial increase in the number of people with PDR, which is reported to currently affect approximately 1.7% (range 0.36–13%) of all diabetics [4]. It will not only lead to significant pressures on health services but will have a considerable impact on the socioeconomic status of individuals affected as well as society in general [5]. Early detection and timely treatment has been shown to prevent progression to irreversible severe vision loss (SVL) in these patients [6, 7].

The landmark Diabetic Retinopathy Study (DRS), concluded over three-decades ago that laser photocoagulation reduces the risk of severe visual loss by 50% or more in eyes with proliferative disease with or without high risk characteristics [6]. Since then, there has been significant advancement in the screening, referral, and management of these patients, especially in the developed world [8]. However, there is a lack of contemporary evidence on the long-term outcomes of these patients after referral from a population level digital screening service, with the likely detection of early stage disease.

Therefore, this multi-centre study aimed to determine real-world long-term outcomes of patients referred from diabetic eye screening programmes (DESP) with predominantly asymptomatic PDR identified through systematic screening utilising digital photography rather than opportunistic screening or cases detected through symptomatic presentations. In addition, our analysis encompasses not only ocular factors but also systemic variables, aiming to identify associations with poorer outcomes in this specific patient population.

## Methods

### Study population

This was a retrospective study of hospital case notes and screening records. We included all patients referred to adjacent specialist ophthalmology units with PDR over a 36-month period, 2007–9 from two DESPs in the UK, namely the South of Tyne and Liverpool programmes. In 2009 they served total populations of 434,100, and 455,993 respectively, and during the study period 35,635 and 36,908 patients respectively were screened in the programmes with diabetes. The two areas serve similar populations in terms of socioeconomic profile [9]. The baseline demographic and diabetes characteristics of both cohorts were similar (supplementary table A1) and we therefore combined the data from both programmes for statistical analysis and presentation of results. Patients underwent two 45^o^ field digital imaging (macula and disc centred) per eye after dilation of pupils using non-mydriatic camera as per DESP guidelines [10]. All patients were referred based on having probable or definite new vessels on their digital retinal images. We excluded patients who were found to have non-diabetic cause for retinal neovascularisation and those who failed to attend any clinic appointment following referral (supplementary Table A2).

### Data collection

We collected data from clinical notes at the hospital eye services and the screening services records on patients’ demographics, diabetes characteristics (age of onset, duration of diabetes, and treatment at baseline), glycated haemoglobin levels at time of referral (% HbA1c), co-morbidities (hypertension, amputation, kidney disease, ischaemic heart disease, and stroke), best correct visual acuities (BCVA) using early treatment diabetic retinopathy study (ETDRS) letter score, intraocular pressures(IOP), retinopathy grade as per the UK DESP grading classification [11, 12] (namely R0 (no retinopathy)), R1 (background retinopathy), R2 (pre-proliferative retinopathy), R3a (active PDR), and R3s (inactive and treated PDR), lens status and presence of diabetic macular oedema (DMO) at the time of referral (defined as clinically significant macular oedema) [13]. In addition, we extracted information on indications and timings of vitrectomy. Data was collected upon referral at baseline, and up to 10 years of follow-up. Data on co-morbidities was sourced from General Practitioner letters and the endocrinology clinics around baseline assessment. End-stage kidney disease was defined specifically as necessitating dialysis or renal transplant. The patients with active PDR(R3a) were classified as high risk (HR) if showing neovascularization on the disc (NVD)1/3 of a disc diameter or more with or without vitreous/pre-retinal haemorrhage or neovascularization elsewhere (NVE) ½ disc diameter or more with vitreous/pre-retinal haemorrhage [6]. All other patients with only NVEs or small NVD were considered to be low risk (LR) patients [6]. All patients with HR or LR PDR underwent full pan-retinal photocoagulation as defined by the DRS study. Patients with DMO received macular laser alone until the availability of intravitreal Ranibizumab in 2013.

### Study outcomes

The critical outcomes included were severe vision loss (SVL), and the need for vitrectomy in the affected eye. SVL was defined as BCVA of less than 20 ETDRS letter scores (equivalent to a Snellen visual acuity of 3/60). The other important outcomes assessed included patient survival time, and moderate vision loss (MVL) defined as BCVA of less than 70 ETDRS letters score ( ~ equivalent to the legal limit for driving and a Snellen visual acuity of 6/12) in the affected eye.

### Statistical analysis

IBM® SPSS version 23 for Windows was used to conduct statistical analyses. All continuous variables were normally distributed and compared between groups using analysis of variance (ANOVA). The Chi-square test was used to compare categorical variables between retinopathy groups. Numerical data were presented as mean with standard deviation (SD), and frequencies with percentages were calculated for categorical variables.

The Cox-regression method was used to analyse survival outcomes. One outcome, death, was observed at the patient level, rather than the eye-level and thus only included one observation per patient. The other outcomes (vitrectomy, blindness, sight impairment) were eye-level outcomes, and so one observation per eye was included in the analysis. For these outcomes, it is possible that outcomes of two eyes from the same patient may be more similar than those of two eyes from different patients. Thus, to allow for this, the regression methods for these outcomes were performed with robust standard errors. This allowed the observations to be independent across patients, but not necessarily within patients.

For all outcomes, regression analyses were performed in two stages. Initially, the separate association between each factor and the outcome was examined in a series of univariable analyses. Subsequently, univariate associations with *p* < 0.2 were examined in a multivariable analysis. A backward selection procedure was performed to retain only the significant factors in the final model. The hazard ratios (HR) with 95% confidence intervals (CI) were calculated and presented for factors with a significant association.

## Results

A total of 150 patients (212 eyes) were referred from DESP during the 36-month study period with a diagnosis of probable or suspected PDR, equating to an annual incidence of approximately 2 per 1000 in the screened diabetic population. Active PDR(R3a) was confirmed in 72 patients (109 eyes) at the initial hospital visit (approximately 1 patient per 1000 of screened population per annum). Eight of these patients had fellow-eyes, which were referred as NPDR but were found to have active R3a during the initial hospital visit. Demographic and baseline characteristics of the patients with R3a in either one or both eyes are summarised in Table 1. The remaining 103 referred eyes were found to have either R1(*n* = 19), R2 (*n* = 61), or R3s (*n* = 23).

**Table 1:** Demographic and baseline characteristics (*n* = 64 patients, 109 eyes).

Variable	R3a	LR PDR	HR PDR	*P* - values^α^
No. of eyes	109	71	38	
No. of patients	72	44	28	
Age, years (mean ± SD)	49.6 ± 12.7	49.8 ± 13.6	46.5 ± 12.1	0.43
Female Sex, *n* (%)	25(34.7%)	14 (31.8%)	11 (39.3%)	0.56
HbA1c, % (mean ± SD)	9.8 ± 1.9	10.1 ± 2.1	9.7 ± 1.6	0.69
Diabetes Type 2, *n* (%)	31(48.4%)	24 (54.5%)	14 (50.0%)	0.92
Diabetes duration, years (mean ± SD)	16.6 ± 8.8	17.5 ± 8.9	16.0 ± 9.1	0.93
CI- DMO	24	17 (23.9%)	7 (18.4%)	0.51
Co-morbidities, Yes(%)	57 (79.2%)	36 (81.8%)	21 (75.0%)	0.86
Hypertension, *n* (%)	53 (73.6)	33 (75.0%)	20 (71.4%)	
Digital/Limb Amputation, *n* (%)	7 (9.7%)	5 (11.4%)	2 (7.1%)	
Stroke, *n* (%)	7 (9.7%)	3 (6.8%)	4 (14.3%)	
Ischaemic Heart Disease, *n* (%)	8 (11.1%)	6 (13.6%)	2 (7.1%)	
End-Stage Renal Impairment, *n* (%)	10 (13.9%)	7 (15.9%)	3 (10.7%)	

*LR PDR* Low risk proliferative diabetic retinopathy

*HR PD* High risk proliferative diabetic retinopathy

*SD* standard deviation

*HbA1c* Glycated haemoglobin

*CI- DMO* Centre involving diabetic macular oedema

*α*: *P*-values compare LR and HR PDR patient groups. Each patient contributed one data point based on their worst retinopathy grade if eyes differed. Co-morbidities were analysed as a cumulative binary variable.

Thirty-seven out of 72 patients (51.4%) (74 eyes) had bilateral R3a at presentation. In patients with unilateral R3a (*n* = 35, 48.6%) the fellow eye grade was R1, R2 and stable PDR (R3s) in 4, 23 and 8 patients respectively. Forty-four patients (61.1%) were found to have low risk PDR and the remaining 28 (38.9%) had high risk features in at least one of their eyes at presentation. There were no statistically significant differences found between the age, duration of diabetes and HbA1c levels between low and high risk R3a groups (Table 1).

### Visual outcome

Out of total 109 eyes with R3a at baseline, eight (7.3%) developed severe vision loss (SVL). High risk eyes had a higher incidence of SVL compared to low-risk eyes with 5 (13.2%) in the HR PDR and 3 (4.2%) in the LR PDR group developing SVL by the study end (*p* = 0.20). Similarly, MVL occurred in a total of 16 (14.7%) eyes, and again the incidence was higher amongst the HR PDR (18.4%) compared to the LR PDR (12.7%) group although without statistical significance difference (*P* = 0.55). Out of the 16 eyes affected by MVL, six eyes were the better seeing ones for the patients. Univariate regression analyses suggested that none of the analysed variables were significantly associated with sight impairment (supplementary Table A3).

The mean best-corrected ETDRS letter score vision for all R3a eyes at presentation and final follow-up was 76.5 ± 14.4 and 65.7 ± 19.0 respectively. The difference was statistically significant (*p* < 0.001). However, we did not find any significant differences in final BCVA between HR PDR and LR PDR groups (*p* = 0.19).

The causes, for SVL included diabetic maculopathy including ischaemic maculopathy (*n* = 3), tractional retinal detachment with macular atrophy (*n* = 2), end-stage rubeotic glaucoma (*n* = 1), chronic vitreous haemorrhage in patient who refused surgery (*n* = 1), and central retinal artery occlusion (*n* = 1). There was only one patient who developed binocular severe sight impairment (SSI) due to acquired bilateral optic atrophy unrelated to his diabetic eye disease.

### Outcome – vitrectomy

**Fig. 1:**
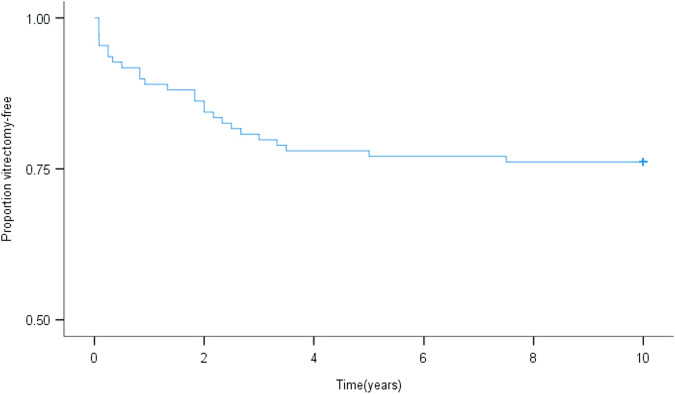
Kaplan-Meier survival curve for outcome of vitrectomy. The plot illustrates vitrectomy-free survival for all R3a eyes over time.

A total of 26 eyes (23.9%) with R3a required vitrectomy. The 10-year ‘survival’ (free from vitrectomy) in all R3a eyes was 76.1% (95% CI: 69.0% to 85.0%). Similar proportion of eyes required vitrectomy in HR PDR (*n* = 9, 23.7%) and LR PDR (*n* = 17, 23.9%) groups. A more detailed illustration of vitrectomy free survival over time for the R3a eyes is shown in Fig. 1. The univariate regression analyses indicated that none of the factors were significantly associated with the time to vitrectomy (supplementary Table A4).

Vitreous haemorrhage was the most frequent indication for vitrectomy, present in 17 out of 26 eyes requiring vitrectomy (65.4%). This was followed by tractional retinal detachment (*n* = 7, 26.9%), a combination of tractional and rhegmatogenous detachment (*n* = 1, 3.8%), and pre-macular haemorrhage (*n* = 1, 3.8%). Overall, the mean time to vitrectomy from presentation was 20.9 ± 21.2 (range: 0–90) months. Although the duration was shorter for HR PDR eyes (16.0 ± 12.6) compared to LR PDR eyes (23.4 ± 24.5), the difference was not statistically significant (*p* = 0.41).

The mean BCVA pre-operatively was 42.2 (standard deviation (SD) 29.2) compared to 66.2 (SD 20.0) ETDRS letter score post-operatively (*p* = 0.005).

### Outcome – death

**Fig. 2:**
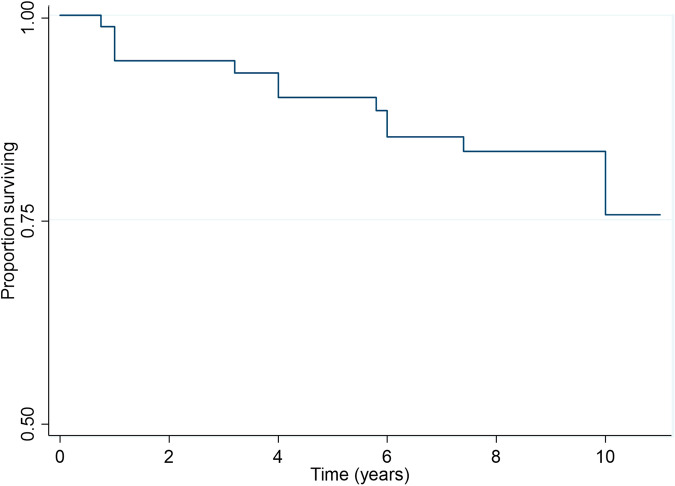
Kaplan-Meier survival curve for outcome of death. The plot illustrates the proportion of all R3a patients surviving over time.

The 10-year survival in all R3a patients was 76.0% (95% CI: 63.0% to 85.0%). Figure 2 illustrates the survival over time for the patients with R3a at presentation.

Univariate analyses found type of diabetes and age to be significantly associated with survival time (supplementary Table A5).

Multivariable analysis however showed that only age was significantly associated with the survival times of the R3a patients. The risk of death at any time was four times higher in those aged 50 years or over compared to the under 50 years group (95% CI: 1.33 to 13.3, *p* = 0.008) (supplementary Fig. 1). A graphical illustration of this result is shown in supplementary Fig. 1. More deaths were observed in the patients with HR PDR (*n* = 10, 26.3%) compared to patient with LR PDR (*n* = 14, 19.7%). However, the regression analysis did not find PDR subtype to have any statistically significant association with patient 10-year survival (95% CI: 0.57 to 4.31, *P* = 0.39). The mean time to death for all deceased patients was 5.37 (SD 3.63) years.

### Outcome – conversion to R3a in fellow eye

**Fig. 3:**
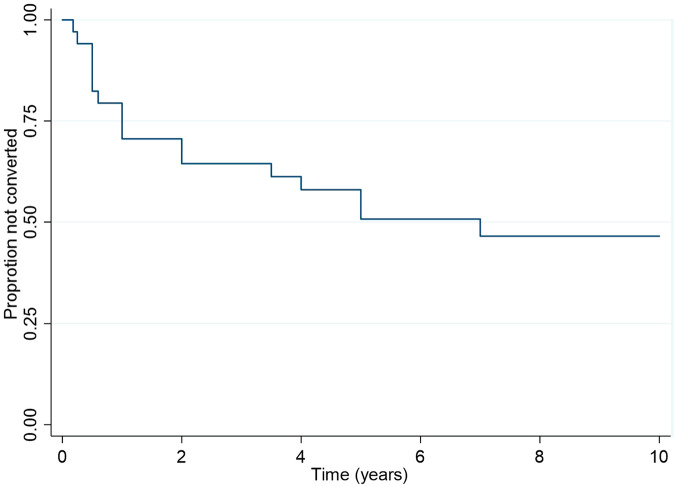
Kaplan-Meier survival for outcome conversion to R3a in fellow eye. The plot illustrates the proportion of fellow non-R3a eyes of patients with unilateral PDR that did not progress to active PDR (R3a) over time.

Thirty-five patients had unilateral R3a at baseline, and we analysed conversion of the fellow non-R3a eyes (*n* = 35) to active PDR. Almost half (*n* = 17; 48.6%) of these fellow eyes converted to R3a during 10-year follow-up with mean time to conversion 2.0 (SD 2.1) years (Fig. 3). Eight (22.9%) other R2 fellow eyes received prophylactic laser photocoagulation. Three fellow eyes required vitrectomy during follow-up at 2, 3 and 9.5 years respectively.

The results of the univariable analyses suggested that diabetes type and age were significantly associated with the time to conversion to R3a (supplementary Table A6). Type I diabetics and younger patients ( < 50 years) were more likely to convert to R3a during follow-up. However, multivariable analysis results suggested that neither were independently associated with the outcome. Other variables including presence of comorbidities, fellow eye retinopathy grade and PRP were not significantly associated with the outcome.

## Discussion

This study highlights the key outcomes of eyes referred with active proliferative diabetic retinopathy from the UK digital photography based diabetic retinopathy screening programme in two UK centres. The estimated incidence rate of PDR per screened diabetics in our study population was similar to the rate of 0.1% reported by a large UK population based study [14]. In our study, just under half of the referred R3a eyes from DESP actually had an active proliferative disease confirmed at their initial hospital visit. Eight eyes were diagnosed with NPDR by the screening programme and were found to have R3a at the baseline. It’s possible that these eyes may have converted to R3a while waiting to be seen in the hospital eye service, but it does highlight the challenges in accurately grading R3a on fundal photography alone. Pre proliferative retinopathy (R2) with coarse intraretinal microvascular abnormalities (IRMA) was the commonest grade confused with R3a followed by treated/stable proliferative disease (R3s). The likely explanation for this is the inherent limitation of 2D digital photographs to reliably differentiate between IRMA and new vessels, as well as the difficulty in assessing the stability of treated new vessels and the completeness of scatter laser without wide field imaging [15–18]. Additional wide field imaging to assess the extent of laser and residual new vessels at discharge from hospital eyes services could help reduce unnecessary referrals in patients with R3s. Similarly, OCT to help differentiate IRMA from actual new vessels that extend through the ILM may also be of utility [19].

We found that patients with active PDR (R3a) had high baseline HbA1c levels. Several studies have linked higher incidence of PDR in patients with poor glycaemic control usually quantified by serum glycated haemoglobin levels(HbA1c) [20–25]. Patients with levels greater than 9.0% are reported to have over two-fold increased risk of progressing to PDR, and the impact is compounded by younger age of diabetes onset [21, 25]. The Wisconsin modelling study estimated that for every 1.0% increment in baseline HbA1c there was nearly double the risk for progressing to PDR (odds ratio 1.86 [95% CI :1.67–2.08]) [21]. Approximately two thirds of the patients had low risk PDR, and one third high risk. The cohort of HR PDR patients was relatively younger and had higher mean HbA1c baseline level compared to the patients with LR PDR, although the differences were not statistically significant.

Although men were more commonly affected in both groups of PDR, the male to female ratio remained almost equally distributed, and the differences were statistically insignificant (*P* = 0.56). Both epidemiological studies from around the globe as well as prognostic studies have reported no discernible differences in the prevalence or rates of progression to PDR between males and females [21, 26–29].

### Visual outcome

One in five R3a eyes during follow-up developed sight impairment falling below UK driving standards (visual acuity worse than 70 letters or <6/12), despite prompt treatment at baseline. During follow-up, eyes lost a mean of over 10 letters of vision compared to baseline. The main reasons to account for this visual loss was centre involving DMO (*n* = 9; 40.9%) followed by cataract (*n* = 5; 22.7%). The ETDRS follow-up study in 2002 reported long-term (13 to 19.5 years) incidence of significant vision loss (less than 20/40 in patients’ better eye) to be 16.0%, which is comparable to our study [30]. They also reported that none of their patients developed SVL. In contrast, eight (7.3%) eyes in our study suffered with SVL mainly due to late complications of PDR. A possible explanation for this difference is that ETDRS follow-up study only included those patients who were alive at the time of the long-term follow-up assessment, and this selection bias means that patients with the worst visual acuity were the least likely to survive up-to the time of follow-up assessment [30]. Moreover, the ETDRS study reported binocular visual outcomes using acuities from the better seeing eye, and additionally included patients with severe NPDR at baseline. A recent study using a large ophthalmology registry estimated the probability of sustained blindness (VA < 20/200) to be 3.2% for eyes with PDR at 2-years [31]. Extrapolating this risk would likely reveal similar rates of SVL after long-term follow-up compared to our study.

The landmark ETDRS study concluded that eyes with high risk characteristics were more likely to develop SVL [20]. Although, we observed that a higher percentage of eyes with HR PDR developed SVL (*n* = 5 ; 13.2%) compared to LR PDR (*n* = 3; 4.2%), the results did not reach statistical significance probably due to the relatively small sample size.

### Outcome – death

Several studies have concluded increased risk of all-cause mortality in patients with proliferative diabetic retinopathy, and reported long-term survival rates in a wide range from 68.0–91.0% [32–36]. We estimated the 10-year survival to be 76.0% which is comparable to recent studies [35, 36]. Older age at the time of presentation was the only significant risk factor associated with increased mortality. This is consistent with other studies who in addition reported chronic renal failure and limb ulcers as significant prognostic factors for mortality as well [30, 35, 36].

### Outcome – vitrectomy

The Diabetic Retinopathy Vitrectomy Study (DRVS) provided evidence in support of early vitrectomy in type 1 diabetics [37]. Since, the landmark DRVS study there has been significant advancement in surgical techniques [38] and use of perioperative anti-vascular endothelial growth factor (anti-VEGF) [39] has led to improved outcomes. As a result of this, it is common observation in routine practice that the rate of vitrectomy has increased despite screening, likely due to a lowered threshold for surgery [40]. The ETDRS study reported 5-year vitrectomy rate of 2.1% in the group assigned to early laser photocoagulation and 4.0% in the deferred treatment group [41]. We found the rate of vitrectomy to be significantly higher (28.3%) in our cohort of patients. Our results are comparable to a recent study from India who found that 31.4% of eyes required vitrectomy during 10-year follow-up [42]. The same study also reported that eyes with low-risk PDR were less likely to require vitrectomy. However, in this study we could not find any significant differences in rates of vitrectomy between the HR and LR PDR groups. Vitreous haemorrhage remained the most common indication for vitrectomy in all studies including ours. Our data also suggests that eyes that do require vitrectomy, do so within 3 years of presentation highlighting the need for close observation during this period. Furthermore, in our study only three non-R3a at baseline fellow eyes required vitrectomy with the rate (11.1%) significantly lower compared to other studies who have reported rate of vitrectomy in fellow eyes ranging from 24.0% to 38.0% with up to 5-years follow-up [43–45]. This substantial difference in rates could be due to the relatively smaller sample size of non-R3a fellow eyes in our study as well as differences in patient characteristics between study populations. We also speculate that prophylactic laser photocoagulation carried out in almost one-third of fellow eyes in our study may have protected against the development of complications of PDR requiring vitrectomy.

### Strengths and limitations

The key strength of our study was the 10-year follow-up of patients with R3a in two tertiary care hospital settings. All surviving patients had complete 10-years follow-up data available. We accept however that the study has several limitations including its retrospective design. We were unable to gather data on comorbidities for a significant number of patients which prevented us from analysing the association of individual comorbidities with survival of these patients. Secondly, we could not determine the precise cause of death in some patients, including how many were diabetic related. Moreover, our sample size was relatively small, especially when divided into the subgroups of LR and HR PDR limiting our ability to show significant differences. Lastly, our locations shared a similar socioeconomic background largely composed of white British patients, making our study less generalisable to other populations.

## Conclusion

Our study provides UK real-world long-term outcomes of patients referred with PDR from systematic diabetic retinopathy screening programmes. Severe vision loss was not a common occurrence at 7% of eyes, but nearly one-fifth of the eyes fell below UK driving standards for vision. Vitrectomy is an important intervention for managing these patients, required in approximately 25% of eyes, and close observation is recommended especially during the initial 3 years following diagnosis for any complications and conversion of fellow-eyes to PDR. The outcomes of this study can assist any future risk-modelling studies to predict outcome in PDR patients.

## Summary

### What was known before

- The number of patients with diabetes is expected to rise significantly in the coming years
- Proliferative diabetic retinopathy (PDR) is an important cause of severe visual impairment
- Early detection and prompt treatment can prevent irreversible sight impairment and visual disability.

### What this study adds

- Severe visual impairment was not a common finding
- Significant number of patients had moderate vision loss falling below UK driving standards
- Vitrectomy was required in one-fourth of the eyes with PDR

## Supplementary information

Electronic Supplementary Material

## Data Availability

The data that support the findings of this study are available from the corresponding author upon reasonable request.
