# Supplementary material for: Referrals for proliferative diabetic retinopathy from two UK diabetic retinopathy screening services: a 10-year analysis of visual outcomes, requirement for vitrectomy, and mortality
Source: Eye (Lond). 2024 Apr 23;38(13):2561–7. doi: 10.1038/s41433-024-03078-1 (PMC11385226; doi:10.1038/s41433-024-03078-1)
Supplement: Supplementary file 1 — Electronic Supplementary Material [file 41433_2024_3078_MOESM1_ESM.pdf]

Electronic Supplementary Material

**Supplementary Table A1**: Baseline Characteristics		
	Sunderland Dataset	Liverpool Dataset
**Total Patients** referred from DESP with probable PDR	94	56
**Demographics**		
Age, years (mean ± SD, (range))	55.1 ± 15.1, (19 - 92)	47.5 ± 13.9, (20-77)
Female Sex, n (%)	36(38.3%)	20(35.7%)
Caucasian, n (%)	91(96.8%)	54(96.4%)
Diabetes Type 2, n (%)	57(60.6%)	34(60.7%)
Diabetes duration, years (mean ± SD, (range))	18.2 ± 9.4, (1 - 42)	15.7 ± 9.5, (0 - 43)
HbA1c, % (mean ± SD)	9.1 ± 1.7 %	9.5 ± 2.2%
**Co-morbidities at baseline**		
Hypertension, n (%)	72(76.6%)	41(73.2%)
Digital/Limb Amputation, n (%)	4(4.3%)	4(7.1%)
Stroke, n (%)	11(11.7%)	4(7.1%)
Ischaemic Heart Disease, n (%)	16(17.0%)	5(8.9%)
End-Stage Renal Impairment, n (%)	5(5.3%)	2(3.6%)
**Management of diabetes**		
Diet only, n (%)	2(2.1%)	1(1.8%)
Oral drugs only, n (%)	26 (27.7%)	19(33.9%)
Insulin only, n (%)	37(39.4%)	22(39.3%)
Combined, n (%)	29(30.9%)	14(25.0%)

SD: standard deviation
DESP: Diabetic eye screening program
PDR: Proliferative diabetic retinopathy
HbA1c: Glycated haemoglobin

**Supplementary Table A2:** Reasons for exclusion	
**Reasons for exclusion**	**Number of patients (n)**
Retinal vein occlusion with disc collaterals	19
New vessels secondary to Ocular Ischemic Syndrome	1
New vessels secondary to retinal artery occlusion	2
Epiretinal membrane causing retinal vessel tortuosity mistaken for a new vessel	1
Refused to attend hospital appointment	2
Died before being assessed in the hospital	4
Relocated after referral with no follow-up available	2

Supplementary Table A3: Univariate analyses examining baseline variables associated with moderate vision loss(MVL)

Variable	Category	MVL n/N (%)	Odds Ratio (95% CI)	P-value
Diabetes type	Type I<br>Type II	10/55 (18%)<br>12/54 (22%)	1<br>1.29 (0.41, 4.02)	0.67
HbA1c	≤ 8.5%<br>> 8.5%	3/24 (13%)<br>14/71 (20%)	1<br>1.72 (0.32, 9.19)	0.53
Duration DM (*)	-	-	0.94 (0.59, 1.50)	0.79
Age	< 50<br>50 +	11/60 (18%)<br>11/49 (22%)	1<br>1.29 (0.41, 4.05)	0.66
Gender	Male<br>Female	15/68 (22%)<br>7/41 (17%)	1<br>0.73 (0.22, 2.37)	0.60
R grade (fellow)	R1 / R2<br>R3a LR<br>R3a HR<br>Rs	8/27 (30%)<br>8/45 (18%)<br>4/29 (14%)<br>2/8 (25%)	1<br>0.51 (0.14, 1.92)<br>0.38 (0.08, 1.75)<br>0.79 (0.13, 4.85)	0.60
R grade (hospital)	R3a LR<br>R3a HR	13/71 (18%)<br>9/38 (24%)	1<br>1.38 (0.47, 4.07)	0.55
CI-DMO	No<br>Yes	14/85 (16%)<br>8/24 (33%)	1<br>2.54 (0.71, 9.06)	0.15
Vitreous haemorrhage	No<br>Yes	20/102 (20%)<br>2/7 (29%)	1<br>1.64 (0.30, 8.38)	0.57
Comorbidities	No<br>Yes	6/18 (33%)<br>16/91 (18%)	1<br>0.43 (0.11, 1.70)	0.23
Follow-up (Years)	-	-	1.06 (0.89, 1.28)	0.51

(*) Odds ratio given for a 5-year increase in diabetes duration
MVL: Moderate vision loss
DM: Diabetes Mellitus
CI-DMO: Centre involving diabetic macular oedema
LR PDR: Low risk proliferative diabetic retinopathy
HR PD: High risk proliferative diabetic retinopathy
CI: Confidence Interval
HbA1c: Glycated haemoglobin

Supplementary Table A4: Univariate analyses examining baseline variables associated with time to vitrectomy

Variable	Category	10-yr vitrectomy free % (95% CI)	Hazard Ratio (95% CI)	P-value
Diabetes type	Type I	77% (63%, 86%)	1	0.58
	Type II	71% (56%, 82%)	1.30 (0.52, 3.23)	
HbA1c	≤ 8.5%	81% (56%, 92%)	1	0.37
	> 8.5%	69% (57%, 79%)	1.73 (0.52, 5.71)	
Duration DM (*)	-	-	1.06 (0.88, 1.27)	0.55
Age	< 50	74% (60%, 83%)	1	0.80
	50 +	76% (60%, 86%)	0.89 (0.36, 2.19)	
Gender	Male	76% (63%, 84%)	1	0.90
	Female	72% (54%, 84%)	1.06 (0.42, 2.66)	
R grade (fellow)	R1 / R2	75% (51%, 88%)	1	0.67
	R3a LR	75% (59%, 85%)	1.53 (0.43, 3.59)	
	R3a HR	77% (56%, 89%)	1.06 (0.36, 3.15)	
	R3s	60% (20%, 85%)	2.35 (0.57, 9.75)	
R grade (hospital)	R3a LR	75% (62%, 83%)	1	0.88
	R3a HR	74% (56%, 86%)	1.06 (0.47, 2.39)	
CI-DMO	No	76% (65%, 84%)	1	0.49
	Yes	70% (47%, 85%)	1.43 (0.51, 4.00)	
Vitreous haemorrhage	No	75% (65%, 82%)	1	0.69
	Yes	69% (21%, 91%)	1.33 (0.32, 5.52)	
Comorbidities	No	89% (62%, 97%)	1	0.42
	Yes	71% (61%, 80%)	2.33 (0.30, 17.8)	

(*) Odds ratio given for a 5-year increase in diabetes duration
DM: Diabetes Mellitus
CI-DMO: Centre involving diabetic macular oedema
LR PDR: Low risk proliferative diabetic retinopathy
HR PDR: High risk proliferative diabetic retinopathy
CI: Confidence Interval
HbA1c: Glycated haemoglobin

Supplementary Table A5: Univariate analyses examining factors associated with survival times

Variable	Category	10-yr survival % (95% CI)	Hazard Ratio (95% CI)	P-value
Diabetes type	Type I	87% (69%, 95%)	1	**0.04**
	Type II	63% (43%, 78%)	3.34 (1.06, 10.5)	
HbA1c	≤ 8.5%	59% (30%, 79%)	1	0.12
	> 8.5%	79% (62%, 89%)	0.43 (0.15, 1.24)	
Duration DM (*)	-	-	0.89 (0.65, 1.21)	0.46
Age	< 50	88% (71%, 95%)	1	**0.008**
	50 +	60% (38%, 76%)	4.20 (1.33, 13.3)	
Gender	Male	77% (60%, 87%)	1	0.59
	Female	73% (49%, 87%)	1.33 (0.47, 3.73)	
R grade (fellow)	R1 / R2	82% (59%, 93%)	1	0.62
	R3a LR	75% (52%, 88%)	1.53 (0.43, 5.44)	
	R3a HR	64% (24%, 87%)	2.25 (0.50, 10.1)	
	Rs	70% (22%, 92%)	2.76 (0.50, 15.2)	
R grade (hospital)	R3a LR	79% (62%, 89%)	1	0.39
	R3a HR	70% (46%, 85%)	1.56 (0.57, 4.31)	
CI-DMO (+)	No	76% (62%, 86%)	1	0.87
	Yes	73% (38%, 91%)	0.90 (0.25, 3.19)	
Vitreous haemorrhage (+)	No	75% (61%, 84%)	1	0.84
	Yes	86% (33%, 98%)	0.81 (0.11, 6.18)	
Comorbidities	No	88% (39%, 98%)	1	0.43
	Yes	74% (59%, 84%)	2.26 (0.30, 17.2)	

(*) Odds ratio given for a 5-year increase in diabetes duration
DM: Diabetes Mellitus
CI-DMO: Centre involving diabetic macular oedema
LR PDR: Low risk proliferative diabetic retinopathy
HR PDR: High risk proliferative diabetic retinopathy
CI: Confidence Interval
HbA1c: Glycated haemoglobin

Supplementary Table A6: Cox regression analyses analyses examining factors associated with conversion of fellow non-R3a eyes to R3a(active disease)

Variable	Category	Univariable		Multivariable	
		Hazard Ratio (95% CI)	P-value	Hazard Ratio (95% CI)	P-value
Comorbidities	No	1	0.33		
	Yes	0.56 (0.18, 1.77)			
Type of diabetes	Type II	1	**0.02**	1	0.25
	Type I	3.54 (1.23, 10.2)		2.18 (0.57, 8.38)	
Age	< 50	1	**0.02**	1	0.27
	50 +	0.29 (0.10, 0.82)		0.47 0.12, 1.79)	
Fellow R grade	LR PDR	1	0.69		
	HR PDR	0.79 (0.26, 2.43)			
PRP	No	1	0.26		
	Yes	0.42 (0.10, 1.86)			

LR PDR: Low risk proliferative diabetic retinopathy
HR PDR: High risk proliferative diabetic retinopathy
CI: Confidence Interval
PRP : Pan retinal photocoagulation

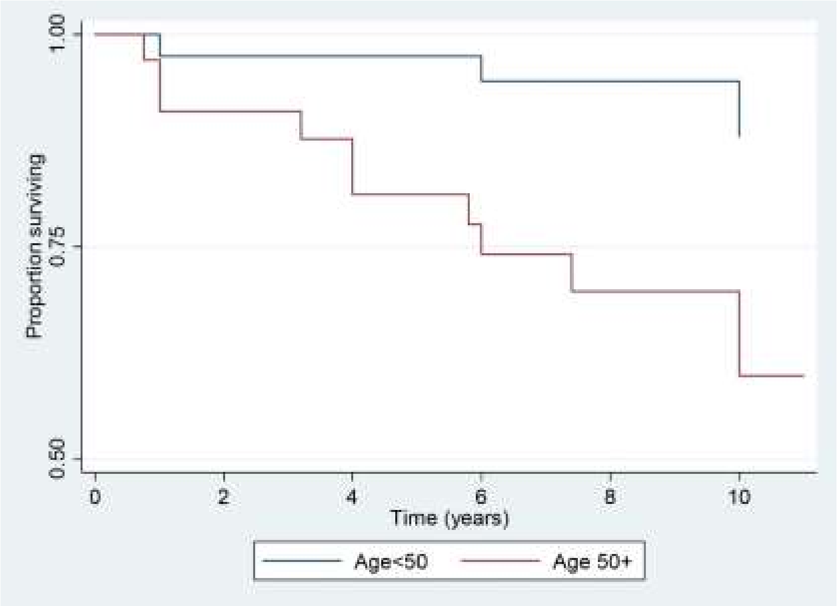

Supplementary Figure 1 - Kaplan-Meier plot of time to death in R3a patients, by age
